# Poly[[μ-1,4-bis­(1*H*-imidazol-4-yl)benzene-κ^2^ *N* ^3^:*N* ^3′^](μ-5-methyl­isophthalato-κ^2^ *O* ^1^:*O* ^3^)cobalt(II)]

**DOI:** 10.1107/S1600536811025657

**Published:** 2011-07-06

**Authors:** Shui-Sheng Chen, Sen-Lin Yang, Shu-Ping Zhang

**Affiliations:** a Department of Chemistry, Fuyang Normal College, Fuyang, Anhui 236041, People’s Republic of China

## Abstract

In the title coordination polymer, [Co(C_9_H_6_O_4_)(C_12_H_10_N_4_)]_*n*_, the Co^II^ atom is four-coordinated by two O atoms from two different 5-methyl­isophthalate bivalent anions and two N atoms from two different 1,4-bis­(1*H*-imidazol-4-yl)benzene ligands, forming a four-coordinated tetra­hedral coordination geometry. Each 5-methyl­isophthalate ligand acts as a μ_2_-bridge, linking two Co^II^ atoms and forming chains which are further linked by 1,4-bis­(1*H*-imidazol-4-yl)benzene ligands into a two-dimensional network parallel to (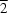
01). These planes are, in turn, linked by two inter­molecular N—H⋯O inter­actions, forming a three-dimensional structure. Weak C—H⋯O hydrogen bonds are also present in the structure.

## Related literature

For background to mixed inorganic-organic hybrid materials, see: Kitagawa & Kondo (1998 ▶). For examples with mixed organic and N-containing ligands, see: Liu *et al.* (2007 ▶); Chen *et al.* (2010 ▶).
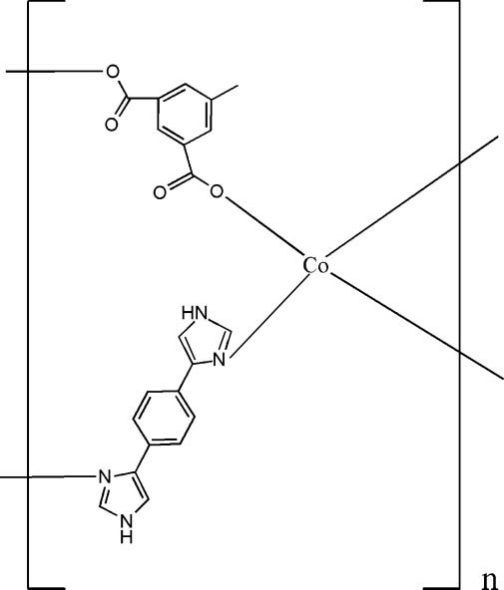


## Experimental

### 

#### Crystal data

[Co(C_9_H_6_O_4_)(C_12_H_10_N_4_)]
*M* *_r_* = 447.31
Monoclinic, 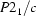

*a* = 7.4608 (5) Å
*b* = 13.8212 (10) Å
*c* = 17.8629 (13) Å
β = 90.451 (1)°
*V* = 1841.9 (2) Å^3^
*Z* = 4
Mo *K*α radiation
μ = 0.97 mm^−1^
*T* = 296 K
0.22 × 0.18 × 0.13 mm

#### Data collection

Bruker SMART APEXII CCD diffractometer
Absorption correction: multi-scan (*SADABS*; Sheldrick, 1996 ▶) *T*_min_ = 0.815, *T*_max_ = 0.884
16427 measured reflections
4210 independent reflections
3540 reflections with *I* > 2σ(*I*)
*R*_int_ = 0.032

#### Refinement

*R*[*F* ^2^ > 2σ(*F* ^2^)] = 0.031
*wR*(*F* ^2^) = 0.100
*S* = 1.12
4210 reflections
272 parameters
H-atom parameters constrained
Δρ_max_ = 0.45 e Å^−3^
Δρ_min_ = −0.34 e Å^−3^

### 

Data collection: *APEX2* (Bruker, 2003 ▶); cell refinement: *SAINT* (Bruker, 2003 ▶); data reduction: *SAINT*; program(s) used to solve structure: *SHELXS97* (Sheldrick, 2008 ▶); program(s) used to refine structure: *SHELXL97* (Sheldrick, 2008 ▶); molecular graphics: *SHELXTL* (Sheldrick, 2008 ▶); software used to prepare material for publication: *SHELXTL*.

## Supplementary Material

Crystal structure: contains datablock(s) global, I. DOI: 10.1107/S1600536811025657/bg2404sup1.cif

Structure factors: contains datablock(s) I. DOI: 10.1107/S1600536811025657/bg2404Isup2.hkl

Additional supplementary materials:  crystallographic information; 3D view; checkCIF report

## Figures and Tables

**Table 1:** Hydrogen-bond geometry (Å, °)

*D*—H⋯*A*	*D*—H	H⋯*A*	*D*⋯*A*	*D*—H⋯*A*
N2—H2*A*⋯O3^i^	0.86	2.16	2.825 (3)	134
N3—H3⋯O2^ii^	0.86	1.96	2.803 (2)	165
C9—H9⋯O2	0.93	2.39	3.274 (3)	158
C11—H11⋯O3^iii^	0.93	2.56	3.182 (3)	124

Symmetry codes: (i) 
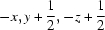
; (ii) 
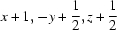
; (iii) 
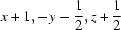
.

**Fig. 1.**
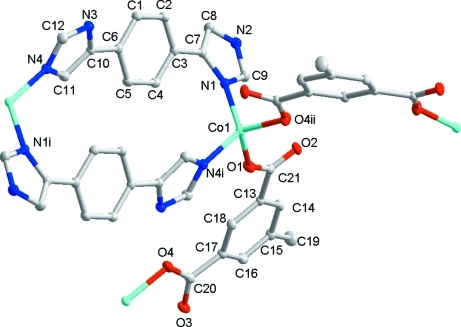
The ORTEP drawing of the title compound (I). Displacement ellipsoids are drawn at 30% probability level. Symmetry codes: (i) 2 - x, -y, 1 - z, (ii) 1 - x, 1/2 + y, 0.5 - z.

**Fig. 2.**
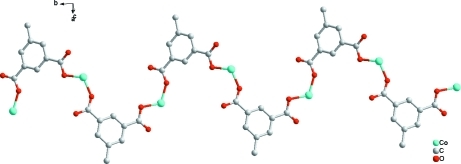
An infinite one-dimensional chain formed from CoII centers and pda2- anions of the compound (I).

**Fig. 3.**
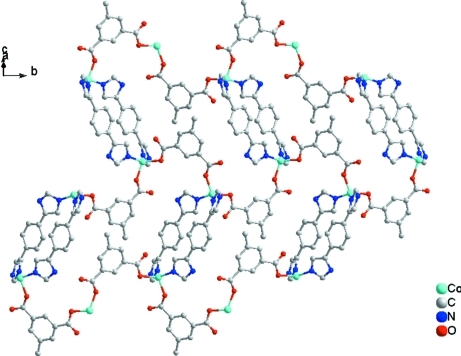
The two-dimensional structure built from one-dimension chains connected by L ligands of the compound (I).

## supplementary crystallographic information

### Comment

In the last decades there has been significant interest in the design and synthesis of mixed inorganic-organic hybrid materials owing to their potential application in catalysis, gas storage and separation, ion exchange and magnetism (Kitagawa & Kondo, 1998)). Recent studies illustrated that mixed organic ligands, especially the mixed polycarboxylate and N-containing ones, with more tunable factors, are good candidates for the construction of novel MOFs (Liu *et al.*, 2007). And based on the mix ligand strategy, we focus our attention in the study of reactions of the 1,4-di(1*H*-imidazol-4-yl)benzene ligand (*L*) together with different carboxylate ligands and salts, and made a systematic investigation on the impact of carboxylate ligands on the structure of the resulting complexes; as a result a series of novel structures have been synthesized (Chen *et al.*, 2010). As an extension of the above work we report herein a new metal complex, Co(C_9_H_6_O_4_) (C_12_H_10_N_4_)]_n_ (I) based on the organic ligands 1,4-di(1*H*-imidazol-4-yl)benzene (*L*) and 5-methylisophthalic acid (H_2_pda), together with Co^II^ salts. In the title compound, the Co ^II^ atom is tetrahedrally coordinated by two nitrogen atoms from two *L* molecules and two carboxylic oxygens atoms from two pda^2-^ ligands (Fig.1). The pda^2-^ ligand acts in a bidentate fashion (via two monodentate carboxylate groups) to connect Co^II^ atoms into a one-dimensional chain (Fig.2), while the *L* ligand acts as a linear bidentate bridge to link chains to form two-dimensional networks paralell to (201) (Fig.3). These planes are in turn linked into a 3D structure by two intermolecular N—H···O interactions and two weaker C-H···O contacts (Table 1)

### Experimental

All reagents and solvents were used as obtained commercially without further purification. A mixture containing CoCl_2_.6H_2_O (23.8 mg, 0.1 mmol), *L* (21.1 mg, 0.1 mmol), DMF (N:*N*'- dimethylformamide, 1 ml), 10 ml H_2_O was sealed in a 16 ml Teflon-lined stainless steel container and heated at 393 K for 72 h. After cooling to room temperature within 12 h, block brown crystals of (I) suitable for X-ray diffraction analysis were obtained in 78% Yield.

### Refinement

H atoms bonded to C atoms were placed geometrically and treated as riding, with C—H distances 0.93 Å and 0.96 Å for aryl and methyl type H-atoms, respectively with *U*_iso_(H) = 1.2*U*_eq_(C) or *U*_iso_(H) = 1.5*U*_eq_(C_methyl_). The amide H atoms were generated theoretically, with the N—H distances 0.86 Å and *U*_iso_(H) = 1.2*U*_eq_(N).

### Crystal data

**Table:**

[Co(C_9_H_6_O_4_)(C_12_H_10_N_4_)]	*F*(000) = 916
*M**_r_* = 447.31	*D*_x_ = 1.613 Mg m^−^^3^
Monoclinic, *P*2_1_/*c*	Mo *K*α radiation, λ = 0.71073 Å
Hall symbol: -P 2ybc	Cell parameters from 5943 reflections
*a* = 7.4608 (5) Å	θ = 2.2–27.5°
*b* = 13.8212 (10) Å	µ = 0.97 mm^−^^1^
*c* = 17.8629 (13) Å	*T* = 296 K
β = 90.451 (1)°	Block, purple
*V* = 1841.9 (2) Å^3^	0.22 × 0.18 × 0.13 mm
*Z* = 4	

### Data collection

**Table:**

Bruker SMART APEXII CCD diffractometer	4210 independent reflections
Radiation source: fine-focus sealed tube	3540 reflections with *I* > 2σ(*I*)
graphite	*R*_int_ = 0.032
phi and ω scans	θ_max_ = 27.5°, θ_min_ = 1.9°
Absorption correction: multi-scan (*SADABS*; Sheldrick, 1996)	*h* = −9→9
*T*_min_ = 0.815, *T*_max_ = 0.884	*k* = −17→17
16427 measured reflections	*l* = −23→23

### Refinement

**Table:**

Refinement on *F*^2^	Primary atom site location: structure-invariant direct methods
Least-squares matrix: full	Secondary atom site location: difference Fourier map
*R*[*F*^2^ > 2σ(*F*^2^)] = 0.031	Hydrogen site location: inferred from neighbouring sites
*wR*(*F*^2^) = 0.100	H-atom parameters constrained
*S* = 1.12	*w* = 1/[σ^2^(*F*_o_^2^) + (0.0533*P*)^2^ + 0.5242*P*] where *P* = (*F*_o_^2^ + 2*F*_c_^2^)/3
4210 reflections	(Δ/σ)_max_ = 0.010
272 parameters	Δρ_max_ = 0.45 e Å^−^^3^
0 restraints	Δρ_min_ = −0.34 e Å^−^^3^

### Special details

**Table:**

Geometry. All e.s.d.'s (except the e.s.d. in the dihedral angle between two l.s. planes) are estimated using the full covariance matrix. The cell e.s.d.'s are taken into account individually in the estimation of e.s.d.'s in distances, angles and torsion angles; correlations between e.s.d.'s in cell parameters are only used when they are defined by crystal symmetry. An approximate (isotropic) treatment of cell e.s.d.'s is used for estimating e.s.d.'s involving l.s. planes.
Refinement. Refinement of *F*^2^ against ALL reflections. The weighted *R*-factor *wR* and goodness of fit *S* are based on *F*^2^, conventional *R*-factors *R* are based on *F*, with *F* set to zero for negative *F*^2^. The threshold expression of *F*^2^ > σ(*F*^2^) is used only for calculating *R*-factors(gt) *etc*. and is not relevant to the choice of reflections for refinement. *R*-factors based on *F*^2^ are statistically about twice as large as those based on *F*, and *R*- factors based on ALL data will be even larger.

### Fractional atomic coordinates and isotropic or equivalent isotropic displacement parameters (Å^2^)

**Table:**

	*x*	*y*	*z*	*U*_iso_*/*U*_eq_	
Co1	0.54880 (4)	0.195950 (18)	0.350426 (14)	0.02058 (10)	
C1	0.7795 (3)	0.12914 (15)	0.66061 (11)	0.0255 (4)	
H1	0.8186	0.1489	0.7078	0.031*	
C2	0.6298 (3)	0.17243 (15)	0.62840 (11)	0.0266 (4)	
H2	0.5685	0.2200	0.6547	0.032*	
C3	0.5697 (3)	0.14555 (14)	0.55695 (11)	0.0226 (4)	
C4	0.6616 (3)	0.07175 (14)	0.52012 (11)	0.0238 (4)	
H4	0.6235	0.0524	0.4728	0.029*	
C5	0.8076 (3)	0.02726 (14)	0.55266 (11)	0.0241 (4)	
H5	0.8643	−0.0229	0.5275	0.029*	
C6	0.8721 (3)	0.05623 (14)	0.62299 (10)	0.0218 (4)	
C7	0.4104 (3)	0.18914 (13)	0.52141 (11)	0.0231 (4)	
C8	0.2542 (3)	0.21709 (16)	0.55384 (12)	0.0295 (5)	
H8	0.2285	0.2165	0.6047	0.035*	
C9	0.2298 (3)	0.23532 (15)	0.43242 (11)	0.0268 (4)	
H9	0.1822	0.2503	0.3856	0.032*	
C10	1.0349 (3)	0.00981 (14)	0.65210 (10)	0.0220 (4)	
C11	1.1089 (3)	−0.07682 (14)	0.63344 (11)	0.0244 (4)	
H11	1.0566	−0.1220	0.6015	0.029*	
C12	1.2952 (3)	−0.00842 (15)	0.70919 (11)	0.0279 (4)	
H12	1.3949	0.0041	0.7392	0.033*	
C13	0.1442 (3)	0.10168 (13)	0.19263 (10)	0.0219 (4)	
C14	−0.0230 (3)	0.11928 (14)	0.16014 (11)	0.0248 (4)	
H14	−0.0810	0.1775	0.1697	0.030*	
C15	−0.1051 (3)	0.05121 (15)	0.11349 (11)	0.0265 (4)	
C16	−0.0144 (3)	−0.03479 (15)	0.09966 (11)	0.0270 (4)	
H16	−0.0651	−0.0798	0.0671	0.032*	
C17	0.1502 (3)	−0.05523 (14)	0.13329 (11)	0.0230 (4)	
C18	0.2293 (3)	0.01403 (14)	0.17953 (11)	0.0234 (4)	
H18	0.3399	0.0015	0.2018	0.028*	
C19	−0.2909 (3)	0.06834 (19)	0.08092 (15)	0.0419 (6)	
H19A	−0.3309	0.0110	0.0554	0.063*	
H19B	−0.2868	0.1213	0.0462	0.063*	
H19C	−0.3724	0.0835	0.1206	0.063*	
C20	0.2386 (3)	−0.15191 (15)	0.12306 (11)	0.0251 (4)	
C21	0.2327 (3)	0.17620 (14)	0.24256 (11)	0.0237 (4)	
N1	0.3928 (2)	0.20055 (12)	0.44391 (9)	0.0231 (4)	
N2	0.1423 (3)	0.24621 (13)	0.49691 (10)	0.0295 (4)	
H2A	0.0348	0.2677	0.5018	0.035*	
N3	1.1559 (2)	0.05179 (12)	0.70134 (9)	0.0258 (4)	
H3	1.1438	0.1069	0.7231	0.031*	
N4	1.2734 (2)	−0.08794 (12)	0.66891 (9)	0.0244 (4)	
O1	0.3807 (2)	0.14966 (11)	0.27237 (9)	0.0342 (4)	
O2	0.1604 (2)	0.25623 (10)	0.25192 (9)	0.0353 (4)	
O3	0.1867 (2)	−0.21003 (11)	0.07470 (9)	0.0337 (4)	
O4	0.3683 (2)	−0.17173 (11)	0.16856 (9)	0.0324 (4)	

### Atomic displacement parameters (Å^2^)

**Table:**

	*U*^11^	*U*^22^	*U*^33^	*U*^12^	*U*^13^	*U*^23^
Co1	0.01940 (16)	0.01927 (15)	0.02303 (15)	0.00030 (10)	−0.00299 (10)	0.00033 (9)
C1	0.0277 (11)	0.0262 (10)	0.0225 (9)	0.0019 (9)	−0.0044 (8)	−0.0028 (7)
C2	0.0287 (12)	0.0236 (9)	0.0273 (10)	0.0052 (9)	−0.0016 (8)	−0.0029 (8)
C3	0.0212 (10)	0.0221 (9)	0.0246 (9)	0.0009 (8)	−0.0017 (7)	0.0037 (7)
C4	0.0232 (10)	0.0266 (10)	0.0216 (9)	−0.0009 (8)	−0.0033 (7)	−0.0009 (7)
C5	0.0231 (10)	0.0253 (10)	0.0240 (9)	0.0030 (8)	−0.0002 (8)	−0.0028 (7)
C6	0.0199 (10)	0.0219 (9)	0.0235 (9)	0.0000 (8)	−0.0016 (7)	0.0036 (7)
C7	0.0249 (11)	0.0207 (9)	0.0237 (9)	0.0000 (8)	−0.0022 (8)	0.0017 (7)
C8	0.0290 (12)	0.0325 (11)	0.0268 (10)	0.0061 (9)	−0.0003 (9)	0.0023 (8)
C9	0.0252 (11)	0.0277 (10)	0.0275 (10)	0.0043 (9)	−0.0028 (8)	0.0032 (8)
C10	0.0210 (10)	0.0239 (9)	0.0211 (9)	0.0003 (8)	−0.0020 (7)	0.0019 (7)
C11	0.0206 (10)	0.0252 (9)	0.0272 (9)	0.0014 (8)	−0.0043 (8)	−0.0016 (8)
C12	0.0241 (11)	0.0303 (10)	0.0292 (10)	0.0013 (9)	−0.0077 (8)	−0.0005 (8)
C13	0.0237 (11)	0.0202 (9)	0.0217 (9)	−0.0015 (8)	−0.0030 (7)	0.0023 (7)
C14	0.0244 (11)	0.0200 (9)	0.0299 (10)	0.0033 (8)	−0.0034 (8)	0.0016 (7)
C15	0.0223 (11)	0.0279 (10)	0.0294 (10)	−0.0009 (9)	−0.0068 (8)	0.0028 (8)
C16	0.0265 (11)	0.0244 (10)	0.0299 (10)	−0.0021 (9)	−0.0071 (8)	−0.0032 (8)
C17	0.0242 (11)	0.0208 (9)	0.0241 (9)	0.0000 (8)	−0.0029 (8)	−0.0008 (7)
C18	0.0210 (10)	0.0227 (9)	0.0264 (9)	0.0007 (8)	−0.0057 (8)	0.0002 (7)
C19	0.0302 (13)	0.0427 (13)	0.0525 (15)	0.0050 (11)	−0.0186 (11)	−0.0027 (11)
C20	0.0234 (11)	0.0227 (10)	0.0291 (10)	−0.0011 (8)	0.0005 (8)	−0.0004 (8)
C21	0.0280 (11)	0.0209 (9)	0.0221 (9)	−0.0014 (8)	−0.0038 (8)	0.0016 (7)
N1	0.0221 (9)	0.0242 (8)	0.0229 (8)	0.0015 (7)	−0.0024 (7)	0.0020 (6)
N2	0.0222 (9)	0.0333 (10)	0.0330 (9)	0.0096 (8)	0.0002 (7)	0.0015 (7)
N3	0.0266 (10)	0.0224 (8)	0.0282 (8)	0.0026 (7)	−0.0059 (7)	−0.0029 (7)
N4	0.0218 (9)	0.0253 (8)	0.0259 (8)	0.0025 (7)	−0.0045 (7)	0.0002 (6)
O1	0.0304 (9)	0.0307 (8)	0.0411 (9)	0.0016 (7)	−0.0150 (7)	−0.0095 (7)
O2	0.0484 (11)	0.0185 (7)	0.0388 (9)	0.0053 (7)	−0.0112 (7)	−0.0024 (6)
O3	0.0338 (9)	0.0255 (7)	0.0419 (9)	−0.0032 (7)	−0.0028 (7)	−0.0104 (6)
O4	0.0343 (9)	0.0277 (7)	0.0352 (8)	0.0100 (7)	−0.0077 (7)	−0.0052 (6)

### Geometric parameters (Å, °)

**Table:**

Co1—O4^i^	1.9611 (15)	C12—N4	1.323 (3)
Co1—O1	1.9744 (15)	C12—N3	1.338 (3)
Co1—N4^ii^	2.0287 (17)	C12—H12	0.9300
Co1—N1	2.0438 (17)	C13—C18	1.388 (3)
C1—C2	1.388 (3)	C13—C14	1.393 (3)
C1—C6	1.397 (3)	C13—C21	1.511 (3)
C1—H1	0.9300	C14—C15	1.395 (3)
C2—C3	1.400 (3)	C14—H14	0.9300
C2—H2	0.9300	C15—C16	1.391 (3)
C3—C4	1.397 (3)	C15—C19	1.517 (3)
C3—C7	1.472 (3)	C16—C17	1.392 (3)
C4—C5	1.375 (3)	C16—H16	0.9300
C4—H4	0.9300	C17—C18	1.392 (3)
C5—C6	1.400 (3)	C17—C20	1.502 (3)
C5—H5	0.9300	C18—H18	0.9300
C6—C10	1.465 (3)	C19—H19A	0.9600
C7—C8	1.361 (3)	C19—H19B	0.9600
C7—N1	1.398 (2)	C19—H19C	0.9600
C8—N2	1.371 (3)	C20—O3	1.239 (2)
C8—H8	0.9300	C20—O4	1.288 (3)
C9—N1	1.323 (3)	C21—O2	1.242 (2)
C9—N2	1.337 (3)	C21—O1	1.276 (3)
C9—H9	0.9300	N2—H2A	0.8600
C10—C11	1.361 (3)	N3—H3	0.8600
C10—N3	1.383 (3)	N4—Co1^ii^	2.0287 (17)
C11—N4	1.385 (3)	O4—Co1^iii^	1.9611 (15)
C11—H11	0.9300		
			
O4^i^—Co1—O1	112.31 (7)	C18—C13—C21	119.74 (18)
O4^i^—Co1—N4^ii^	116.69 (7)	C14—C13—C21	120.84 (18)
O1—Co1—N4^ii^	93.09 (7)	C13—C14—C15	121.23 (19)
O4^i^—Co1—N1	107.14 (7)	C13—C14—H14	119.4
O1—Co1—N1	102.97 (7)	C15—C14—H14	119.4
N4^ii^—Co1—N1	122.64 (7)	C16—C15—C14	118.06 (19)
C2—C1—C6	120.74 (18)	C16—C15—C19	120.63 (19)
C2—C1—H1	119.6	C14—C15—C19	121.3 (2)
C6—C1—H1	119.6	C15—C16—C17	121.72 (19)
C1—C2—C3	120.96 (19)	C15—C16—H16	119.1
C1—C2—H2	119.5	C17—C16—H16	119.1
C3—C2—H2	119.5	C16—C17—C18	118.98 (18)
C4—C3—C2	117.93 (19)	C16—C17—C20	120.98 (18)
C4—C3—C7	119.58 (17)	C18—C17—C20	119.98 (18)
C2—C3—C7	122.44 (18)	C13—C18—C17	120.52 (19)
C5—C4—C3	121.17 (18)	C13—C18—H18	119.7
C5—C4—H4	119.4	C17—C18—H18	119.7
C3—C4—H4	119.4	C15—C19—H19A	109.5
C4—C5—C6	121.12 (18)	C15—C19—H19B	109.5
C4—C5—H5	119.4	H19A—C19—H19B	109.5
C6—C5—H5	119.4	C15—C19—H19C	109.5
C1—C6—C5	118.01 (18)	H19A—C19—H19C	109.5
C1—C6—C10	123.85 (17)	H19B—C19—H19C	109.5
C5—C6—C10	118.13 (18)	O3—C20—O4	122.05 (19)
C8—C7—N1	108.41 (19)	O3—C20—C17	121.79 (19)
C8—C7—C3	128.57 (19)	O4—C20—C17	116.14 (17)
N1—C7—C3	122.82 (18)	O2—C21—O1	125.10 (19)
C7—C8—N2	106.62 (19)	O2—C21—C13	119.87 (19)
C7—C8—H8	126.7	O1—C21—C13	115.03 (17)
N2—C8—H8	126.7	C9—N1—C7	105.86 (17)
N1—C9—N2	111.22 (18)	C9—N1—Co1	114.42 (13)
N1—C9—H9	124.4	C7—N1—Co1	139.11 (15)
N2—C9—H9	124.4	C9—N2—C8	107.89 (18)
C11—C10—N3	105.12 (17)	C9—N2—H2A	126.1
C11—C10—C6	129.41 (18)	C8—N2—H2A	126.1
N3—C10—C6	125.24 (18)	C12—N3—C10	107.96 (17)
C10—C11—N4	110.19 (18)	C12—N3—H3	126.0
C10—C11—H11	124.9	C10—N3—H3	126.0
N4—C11—H11	124.9	C12—N4—C11	105.15 (17)
N4—C12—N3	111.57 (18)	C12—N4—Co1^ii^	128.76 (15)
N4—C12—H12	124.2	C11—N4—Co1^ii^	125.59 (13)
N3—C12—H12	124.2	C21—O1—Co1	138.10 (14)
C18—C13—C14	119.42 (18)	C20—O4—Co1^iii^	109.00 (13)
			
C6—C1—C2—C3	−1.2 (3)	C18—C17—C20—O3	−169.7 (2)
C1—C2—C3—C4	2.0 (3)	C16—C17—C20—O4	−165.3 (2)
C1—C2—C3—C7	179.3 (2)	C18—C17—C20—O4	11.9 (3)
C2—C3—C4—C5	−0.5 (3)	C18—C13—C21—O2	177.05 (19)
C7—C3—C4—C5	−177.92 (19)	C14—C13—C21—O2	−3.7 (3)
C3—C4—C5—C6	−1.8 (3)	C18—C13—C21—O1	−3.0 (3)
C2—C1—C6—C5	−1.1 (3)	C14—C13—C21—O1	176.23 (19)
C2—C1—C6—C10	177.6 (2)	N2—C9—N1—C7	−0.4 (2)
C4—C5—C6—C1	2.5 (3)	N2—C9—N1—Co1	−173.18 (14)
C4—C5—C6—C10	−176.18 (19)	C8—C7—N1—C9	0.6 (2)
C4—C3—C7—C8	140.6 (2)	C3—C7—N1—C9	175.72 (18)
C2—C3—C7—C8	−36.7 (3)	C8—C7—N1—Co1	170.50 (16)
C4—C3—C7—N1	−33.6 (3)	C3—C7—N1—Co1	−14.3 (3)
C2—C3—C7—N1	149.1 (2)	O4^i^—Co1—N1—C9	76.95 (16)
N1—C7—C8—N2	−0.5 (2)	O1—Co1—N1—C9	−41.65 (16)
C3—C7—C8—N2	−175.31 (19)	N4^ii^—Co1—N1—C9	−143.96 (14)
C1—C6—C10—C11	159.7 (2)	O4^i^—Co1—N1—C7	−92.4 (2)
C5—C6—C10—C11	−21.6 (3)	O1—Co1—N1—C7	148.99 (19)
C1—C6—C10—N3	−26.5 (3)	N4^ii^—Co1—N1—C7	46.7 (2)
C5—C6—C10—N3	152.1 (2)	N1—C9—N2—C8	0.1 (3)
N3—C10—C11—N4	−1.1 (2)	C7—C8—N2—C9	0.3 (2)
C6—C10—C11—N4	173.60 (19)	N4—C12—N3—C10	−0.6 (2)
C18—C13—C14—C15	−1.3 (3)	C11—C10—N3—C12	1.0 (2)
C21—C13—C14—C15	179.49 (18)	C6—C10—N3—C12	−173.96 (19)
C13—C14—C15—C16	−0.6 (3)	N3—C12—N4—C11	−0.1 (2)
C13—C14—C15—C19	177.2 (2)	N3—C12—N4—Co1^ii^	172.10 (14)
C14—C15—C16—C17	2.6 (3)	C10—C11—N4—C12	0.8 (2)
C19—C15—C16—C17	−175.2 (2)	C10—C11—N4—Co1^ii^	−171.73 (14)
C15—C16—C17—C18	−2.7 (3)	O2—C21—O1—Co1	10.4 (4)
C15—C16—C17—C20	174.58 (19)	C13—C21—O1—Co1	−169.49 (15)
C14—C13—C18—C17	1.2 (3)	O4^i^—Co1—O1—C21	−55.5 (2)
C21—C13—C18—C17	−179.59 (18)	N4^ii^—Co1—O1—C21	−176.1 (2)
C16—C17—C18—C13	0.8 (3)	N1—Co1—O1—C21	59.4 (2)
C20—C17—C18—C13	−176.52 (18)	O3—C20—O4—Co1^iii^	−2.4 (3)
C16—C17—C20—O3	13.1 (3)	C17—C20—O4—Co1^iii^	176.03 (14)

Symmetry codes: (i) −*x*+1, *y*+1/2, −*z*+1/2; (ii) −*x*+2, −*y*, −*z*+1; (iii) −*x*+1, *y*−1/2, −*z*+1/2.

### Hydrogen-bond geometry (Å, °)

**Table:**

*D*—H···*A*	*D*—H	H···*A*	*D*···*A*	*D*—H···*A*
N2—H2A···O3^iv^	0.86	2.16	2.825 (3)	134
N3—H3···O2^v^	0.86	1.96	2.803 (2)	165
C9—H9···O2	0.93	2.39	3.274 (3)	158
C11—H11···O3^vi^	0.93	2.56	3.182 (3)	124

Symmetry codes: (iv) −*x*, *y*+1/2, −*z*+1/2; (v) *x*+1, −*y*+1/2, *z*+1/2; (vi) *x*+1, −*y*−1/2, *z*+1/2.
